# Intramuscular ketamine vs. escitalopram and aripiprazole in acute and maintenance treatment of patients with treatment-resistant depression: A randomized double-blind clinical trial

**DOI:** 10.3389/fpsyt.2022.830301

**Published:** 2022-07-22

**Authors:** Marco Aurélio Cigognini, Alia Garrudo Guirado, Denise van de Meene, Mônica Andréia Schneider, Mônica Sarah Salomon, Vinicius Santana de Alexandria, Juliana Pisseta Adriano, Ana Maria Thaler, Fernando dos Santos Fernandes, Adriana Carneiro, Ricardo Alberto Moreno

**Affiliations:** Mood Disorders Unit (GRUDA), Institute and Department of Psychiatry, School of Medicine, University of São Paulo (FMUSP), São Paulo, Brazil

**Keywords:** ketamine, treatment-resistant depression, N-methyl-D-aspartate receptor antagonist, randomized clinical trial, intramuscular (IM)

## Abstract

**Objective:**

Ketamine, an N-methyl D-aspartate (NMDA) receptor antagonist, can promote rapid action in the management of individuals with treatment-resistant depression (TRD) at sub-anesthetic doses. However, few studies have investigated the long-term use of ketamine administered intravenously (IV) and intranasally (IN). We report the design and rationale of a therapeutic trial for assessing the efficacy, safety, and tolerability of repeated-dose intramuscular (IM) ketamine vs. active treatment (escitalopram and aripiprazole) in TRD patients.

**Methods:**

A comparative, parallel-group, randomized double-blind trial assessing the efficacy, safety, and tolerability of acute (4 weeks) and maintenance (24 weeks) use of IM ketamine (0.75 mg/kg) vs. active control (escitalopram 15 mg and aripiprazole 5 mg) in individuals with moderate-severe intensity TRD (no psychotic symptoms) with or without suicide risk will be conducted. Patients with TRD (18–40 years) will be randomized and blinded to receive ketamine IM or active treatment at a 1:1 ratio for 4 weeks (active treatment) and 24 weeks (maintenance treatment). Subjects will be assessed using clinical scales, monitored for vital signs (VS) after application of injectable medication, and undergo neuropsychological tests. The primary outcome will be changed on the Montgomery-Åsberg Depression Rating Scale (MADRS) during the course of the trial. The study is in running.

**Results:**

This study can potentially yield evidence on the use of IM ketamine in the treatment of depressive disorders as an ultra-rapid low-cost therapy associated with less patient discomfort and reduced use of medical resources, and can elucidate long-term effects on different outcomes, such as neuropsychological aspects.

**Conclusions:**

The trial can help promote the introduction of a novel accessible approach for the treatment of complex disease (TRD) and also allow refinement of its long-term use.

**Clinical trial registration:**

https://clinicaltrials.gov/ct2/show/NCT04234776, identifier: NCT04234776.

## Introduction

In 2015, an estimated 300 million people had depression (4.4% of the world's population) (1). Depression is a common recurrent serious mental disorder associated with morbimortality and a burden of 50 million years lived with disability (YLD), constituting the mental disease with the highest global disability (2, 3). The economic impact of depression on productivity ran into billions of dollars (presenteeism and absenteeism) in terms of Gross National Product (GDP) (4). The disease is the largest contributor to suicide (some 800,000 deaths/year), a figure which is probably underestimated (5, 6).

Depressive disorders are heterogeneous diseases categorized by international systems of disease classification and whose treatment approaches are well-defined (7–10). However, only 1/3 of patients experience symptom remission after the first intervention (11, 12). Therapeutic goals have changed over time, and include symptoms remission, recovery of function, improvement in quality of life, and cognitive remission, as well as new antidepressants called “atypical”, have been developed in recent decades, such as duloxetine, agomelatine, vortioxetine, among others, in an attempt to optimize therapeutic results (13–15).

A number of definitions of treatment-resistant depression (TRD) exist, but there is a consensus on non-response after the use of 2 or more antidepressants of different classes (with adjustment of adherence, dose, and duration of use) (16, 17). Since the introduction of the concept in 1974, numerous studies have investigated treatment strategies adopting different definitions of the meaning of the illness (18). Treatment-resistant depression accounts for 12–20% of depressed patients and ~20% of TRD cases are staged as chronic, with various unfavorable clinical outcomes and both social and economic impacts (19–21).

Although numerous TRD treatment strategies exist, many interventions have limited efficacy, undesirable side effects and are high cost or inaccessible, besides presenting other barriers to implementation (22, 23). A new effective, less invasive treatment could significantly relieve the suffering and anguish of patients and their families compared to complex interventions for TRD, for example, neurostimulation treatments (24).

The clinical effects of ketamine involve anesthetic, analgesic, antidepressant, and anti-inflammatory actions (25). The drug is derived from phencyclidine and was developed in the 1960s (26). It has two enantiomers: S-ketamine and R-ketamine, with the racemic preparation containing concentrations of 1:1 (27). The drug undergoes hepatic biotransformation by the liver into different metabolites, norketamine being the most important, a product of demethylation by the P450 cytochrome that is excreted by the kidneys (28). Readily distributed by the tissues, including the brain, ketamine is very lipid soluble and exhibits plasma protein binding (12%) with a half-life of around 10 min (29). Ketamine is an N-methyl D-aspartate (NMDA) receptor antagonist with additional weaker actions on sigma receptors, as well as noradrenalin, serotonin, and dopamine transporters among others (30, 31). A signaling cascade may play a role in the regulation, and synaptic plasticity of the mammalian target of rapamycin (mTOR), and in mediating the rapid effects of ketamine (32). Effective, rapid antidepressant effects *via* non-conventional mechanisms represent one of the most important breakthroughs in the field of psychiatry over the last 50 years (33, 34).

Berman et al. carried out the first clinical trial using intravenous (IV) ketamine at a dose of 0.5 mg/kg in a small sample, observing a meaningful reduction on the Hamilton Depression Scale (HAM-D) after 3 days (35). Subsequently, a growing number of methodologically-refined studies (involving single IV applications) were conducted (36–39). A systematic review of 22 studies [randomized clinical trials (RCTs) and non-RCTs] involving 629 participants investigating the role of ketamine [IV 0.5 mg/kg for unipolar depression (UD) and bipolar depression (BD)] found a rapid effect in most of the studies reviewed, observing greatest magnitude of effect in RCTs at 210–230 min post-application (40). In another systematic review, McGirr et al. investigated the efficacy of ketamine (IV 0.5 mg/kg) in the treatment of depressive episodes of 73 individuals in parallel arms and 110 in cross-over designs (34 participants with BD and 149 with UD), where the primary outcome was clinical remission of symptoms at 24 h, 3 and 7 days vs. placebo: results at 24 h [Odds Ratio (OR) of 7.06 and number needed to treat (NNT) of 5], at 3 days (OR of 3.86 and NNT of 6) and, at 7 days (OR of 4 and NNT of 6) (41).

The strategy of repeated ketamine infusions has been explored over the past decade (24). Six 12-day infusions [10 patients (non-medicated)] with DRT who previously responded to 1 dose (MADRS ≥ 50%) had a mean (SD) reduction in scores after the last infusion of 85% (12%) (42). S-ketamine IV was administered to 6 depressed patients (6 infusions over 4 weeks) to investigate clinical efficacy (HAMD-21: after 120 min of application) obtaining scores from the first to the last measurement in 5 subjects of 19 → 11, 19 → 10, 35 → 25, 22 → 1, and 21 → 2 (43). Subjects with RDT (*n* = 24) undergoing up to 6 ketamine infusions (IV, 0.5 mg/kg, 3 times a week, 12 days) had a response rate of 70.8% with a mean decrease in score MADRS 2 h after the first infusion (18.9 ± 6.6; *p* < 0.001); sustained during applications (44). A multi-center, double-blind, randomized, placebo-controlled trial of ketamine (IV 0.5 mg/kg) 2 and 3 times weekly confirmed the efficacy of the 2 regimens, with mean change on the MADRS (baseline to day 15, vs. placebo groups [ketamine twice weekly: −18.4 (SD = 12.0); placebo: −5.7 (SD = 10.2); *p* < 0.001; ketamine 3 times weekly: −17.7 (SD = 7.3); placebo: −3.1 (SD = 5.7); *p* < 0.001] (45).

The safety of ketamine has been confirmed for some decades, with anesthetic doses of 4–5 mg/kg intramuscularly (IM) proving effective [93–100% of children (1,022 pediatric cases)] for airways, emesis, or agitation (46). In a small number of patients, the drug can temporarily affect heart rate, and blood pressure and promote myocardial ischemia, although these risks can be attenuated by the use of sub-anesthetic doses and careful patient selection (27, 41, 44, 47). Dissociative states, a common side effect of the drug, are transient (lasting ~40 min, 2 h after use) and are not associated with persistent psychosis or mood swings (48). Of 158 patients given ketamine, 21 (13.3%) withdrew from the study, compared to 10 (7.4%) out of the 135 patients receiving control interventions (OR 1.95, 95%CI 0.86–4.42, *z* = 1.59, *p* = 0.11) (39). Claims that NMDA receptor blocking can cause “brain damage” and that ketamine affects the urinary retract remain the subject of controversy (49). The most common (≥20%) adverse events following the use of the drug include, headache, anxiety, dissociation, nausea, dizziness, and drowsiness on days of the administration, with these effects dissipating within 2 h, while more serious side effects leading to hospitalization or suicide attempts are rare (40, 50, 51). Anti-depressant effects of ketamine are short-lived (days or weeks), although longer-term benefits can be maintained by use of repeat doses, warranting further studies on the prolonged effects of the drug (44, 52). Intranasal (IN) esketamine was approved for use in 2019 by both the U.S. Food and Drug Administration (FDA) and the European Medicines Agency (EMA) (53). Other administration routes have also been investigated: IN, subcutaneous (SC), and oral (54–56).

With 93% bioavailability, the plasma concentration of IM ketamine is linear (mg/kg) (57, 58). A woman with depression and metastatic ovarian cancer experienced rapid remission (MADRS < 7) 1 h after receiving the first injection (1 mg/kg IM ketamine) (59). The same dosing repeated over 10 months (1× per week) kept MADRS scores low over the period (60). In 2 cases of bipolar depression with suicide risk, 4 applications of IM ketamine (0.75 mg) every 2 days, there was a reduction of 75.5%-83.3%-85.7% (case 1) and 71.4%-77.2%-60.8% (case 2) in the BDI, BAI, and BSI, respectively (61). Twenty-seven subjects in 3 parallel groups (9 subjects each) had ketamine administered IM and IV (G1 = 0.5 mg/kg IV, G = 0.5 mg/kg IM, and G3 = 0.25 mg/kg IM) with the HAM-D reduced by 58.86, 60.29, and 57.36%, respectively (62). IM ketamine has a recently similar response as potent ECT in 6–9 sessions for 3 weeks (HAM-D and BDI-suicide ideation) (63).

The anti-depressant efficacy of IM ketamine can offer several advantages: less cost (IN esketamine), less discomfort, ease of administration, and reduced reliance on the care team and resources than IV ketamine. The safety and tolerability of this route can provide greater accessibility and represent an adjunct in the treatment of depression and management of suicide risk. Although, the studies currently available on the IM route for ketamine are scarce and have methodological shortcomings: incipient samples, open studies, administration of few doses, subjective outcomes, no comparison with active substances, varying doses, case studies, etc. (59–64).

## Methods

### Design and study populations

A comparative, parallel-group, randomized, double-blind trial is to be conducted involving an anti-depressant intervention. The experimental group (EG) will use the experimental substance [ketamine IM 0.75 mg/kg, oral placebo (morning), and oral placebo (evening)], while the control group (CG) will use an active treatment administered orally [escitalopram 15 mg (morning), aripiprazole 5 mg (evening) and placebo IM]. The study will be carried out by the Institute of Psychiatry of the University of São Paulo Medical School (HCFMUSP), in conjunction with the Center for Mind Health Studies (NUPE) in the Vale do Itajaí, Santa Catarina state, Brazil. The study is called the KETAMIM project and is Registered Under No. NCT04234776 on the www.ClinicalTrials.gov platform. The study phases and methodological aspects comply with the recommendations of the CONSORT (Consolidated Standard of Reporting Trials) statement and with Good Clinical Practice guidelines (GCP) (65, 66). The study is in running.

The study shall entail 5 phases: (1) Dissemination (P0): patients referred by health professionals, dissemination in the press or online media; (2) Pre-treatment (PI): ~2 weeks for the baseline assessment of candidates for study entry; (3) Therapeutic trial (PII): 4 weeks for ketamine use 3 times weekly and of daily oral medications, as well as placebos for both groups, characterizing acute treatment of the disease; (4) Maintenance (PIII): 24 weeks for weekly ketamine use and maintenance of standard treatment and respective placebos; (5) Post-treatment (PIV): 4 weeks for assessing patients after use of ketamine, maintenance oral medication or placebo.

A total of 88 patients with TRD (≥2 failed trials using anti-depressants, plus ECT as an alternative or otherwise) of moderate-severe intensity [according to criteria of Diagnostic and Statistical Manual of Mental Disorders (DSM-5)], and the diagnosis confirmed by the Portuguese version of the clinical interview of the DSM-IV, will be recruited (67). Patients will be block randomized using a computer-generated list into one of the two interventions (1:1 ratio), EG or CG (68). A nurse blinded to the allocation will administer ketamine or saline IM, placebo orally, or the active treatment, respectively.

### Inclusion and exclusion criteria

**The following individuals will be eligible for study inclusion:** Subjects residing in Itajaí valley (Santa Catarina state, Brazil); adults (age 18–40 years) with a primary diagnosis of TRD based on clinical evaluation and confirmed by the SCID-IV [research version (TRD defined as failure of 2–5 clinical trials with anti-depressants including ECT)]; moderate-severe intensity disease [clinical criteria and/or score ≥14 on the Hamilton Depression Rating Scale (17-item HAM-D)]; with no psychotic symptoms; not presenting an imminent risk of suicide (as indicated by clinical evaluation and HAM-D) and/or murder; with anxiety disorders (secondary); with compensated clinical comorbidities and; literate and able to understand the tasks requested (69). Patients and/or legal guardians must be aware of the nature of the study and give consent by signing the Free and Informed Consent form. All patients will undergo a physical and neurological examination, laboratory tests, and an electrocardiogram (ECG) to ascertain their comorbid clinical and/or uncompensated conditions. **The following individuals will be excluded from the study:** Subjects exhibiting imminent risk of suicide, presenting bipolar spectrum disorders or other diagnosed psychiatric conditions (primary); psychoactive substance dependence within the last year; intellectual deficit; allergy to ketamine; and glaucoma. Fertile women must be in use of a clinically acceptable method of birth control [oral contraceptive and/or condom (only unfollowed topic in GCP)]. In the event of clinical doubt regarding pregnancy, a test for beta-human chorionic gonadotropin (βHCG) hormone will be ordered. Patients who become pregnant during the study period will be excluded and referred for obstetric care. The medication wash-out period required before visit 0 will be 1 week for antipsychotics, antidepressants [except fluoxetine (4 weeks)], and for mood stabilizers. No other medications with psychiatric action will be allowed during the study, except for Lorazepam and Zolpidem for patients in the use of a benzodiazepine or hypnotic agent.

### Study hypotheses

The principal hypothesis of this study is that ketamine IM provides similar efficacy to active treatment in patients with TRD due to a comparison between the experimental intervention (ketamine IM) vs. active treatment (escitalopram and aripiprazole). Other objectives of the study include confirming the safety and tolerability of the use of ketamine IM during acute and maintenance treatment.

### Measures and outcome variables

The study will assess (given in Table 1):

1. Sociodemographic variables: gender, marital status, ethnicity, number of children, income, education, and employment.
2. Clinical characteristics over the life course: clinical and psychiatric comorbidities, smoking, family history of mood disorders, prior pharmacological treatment and ECT, number of mood episodes, previous psychiatric admissions, suicide attempts, currently receiving psychotherapy, and history of sexual, or physical abuse.
3. Clinical history: visits to the emergency room, attempted suicides, or psychiatric hospitalizations.
4. The Montgomery-Åsberg Depression Rating Scale (MADRS) (70, 71): will be applied as a primary outcome measure 3× per week for the first month (PII), 1× per week for 6 months (PIII), and 1× per week (PIV) for 1 month.
5. The Hamilton Rating Scale for Depression (HAM-D) (72, 73): will be applied once in PI to assess symptom severity and again 3× per week for 1 month (PII), 1× per week for 6 months (PIII), and 1× per week (PIV) for 1 month.
6. Vital signs (VS): pulse oximetry, heart monitoring, breathing rate, heart rate, and blood pressure will be checked continuously using non-invasive methods. VS to be monitored 3× per week for the 1st month (PII) and 1× week (PIII) for 2 h continuously after completion of each application.
7. Global Clinical Impression (GCI) scale: this will be applied 3× a week for 1 month (PII), 1× week for 6 months (PIII), and 1× week (PIV) to assess disease severity, global improvement of the condition and hence, treatment efficacy (74).
8. Depression Thoughts Scale (DTS): the DTS will be applied at visit one (V-1), V11, and V-39 to assess for thought distortions (75, 76).
9. Clinician-Administered Dissociative States Scale (CADSS): this will provide a measure of the dissociative effects of ketamine and will be applied 3× per week for the 1st month (PII), and 1× per week for 6 months (PIII), ~1 h after application of injections (77).
10. Young's Mania Rating Scale (YMRS): this scale will be employed in this study to assess manic switching as an adverse effect of the medications and applied 3× week for the 1st month (PII) and 1× week for 6 months (PIII), ~1 h after application of injections (78).
11. Brief Psychiatric Rating Scale (BPRS): Sub-item 12 of the scale will be used for detecting the presence of psychotic symptoms arising after application of ketamine, applied 3× week for 1 month (PII) and 1× week (PIII), ~1 h after application of injections (79).
12. Udvalg Kliniske Undersoegelser Side Effect Rating Scale (UKU-SERS): this will be used to determine the presence of side effects of the medications used and will be applied 3× week for the 1st month (PII) and 1× week (PIII) thereafter, around 1 h after application of injections (79).
13. World Health Organization Quality of Life, brief version (WHOQOL-Bref): this scale will be applied at V-1, V-11, and V-35 (80).
14. Sheehan Disability Scale (SDS): this scale will be applied at V-1, V-11, and V-35 (81).

**Table 1:** Outcome measurements over time.

**Scales**	**Baseline**	**P1**	**End of P1**	**P2**	**End of P2**	**P3**	**P4**
SCID	x						
MADRS		x		x		x	x
HAM-D	x	x		x		x	x
CGI-S	x	x		x		x	x
DTS	x		x		x		
Physical and neurological exams	x						
Clinical tests and ECG	x						
VS		x		x		x	
UKU-SERS		x		x		x	
BPRS-12		x		x		x	
CADSS		x		x		x	
YOUNG		x		x		x	
WHOQOL-Bref							
SDS	x		x		x		x
Neuropsychological assessment	x				x		

SCID, Structured Clinical Interview for DSM Disorders; MADRS, Montgomery–Åsberg Depression Rating Scale; HAM-D, Hamilton Depression Scale; CGI-S, Clinical Global Impression—Severity; DTS, Depressed Thoughts Scale; VS, Vital Signs; UKU-SERS, Udvalg for Kliniske Undersøgelser Side Effect Rating Scale; BPRS-12, Brief Psychiatric Rating Scale; CADSS, Clinician-Administered Dissociative States Scale; YOUNG, Young Mania Rating Scale; WHOQOL, World Health Organization Quality-of-Life scale; SDS, Sheehan Disability Scale.

### Randomization and allocation

Randomization will be based on a computer-generated scheme, balanced by the use of randomly permutated blocks and stratified by the statistician of the Institute of Psychiatry of the FMUSP to receive ketamine IM or active treatment at a 1:1 ratio (82).

### Intervention

Two groups will be formed: a study group and an active control group. The study group will receive dextroketamine chloralhydrate IM (0.75 mg/kg) plus 2 placebo tablets orally, one in the morning and the other at night. The active control group will receive saline solution IM plus one of the alternative therapies for TRD: escitalopram 15 mg (morning) or aripiprazole 5 mg (night) (10). Injections will be applied alternately to gluteal muscles in the external upper quadrants. For 4 weeks (1 month), applications will be applied on Mondays, Wednesdays, and Fridays (giving a total of 12 interventions). For the ensuing 6 months, applications will be weekly (giving a total of 24 interventions). For 4 weeks (1 month), participants will continue to receive the tablets (active treatment or placebo) and be monitored. Safety parameters: vital signs will be monitored continuously by the researchers for 2 h post-application: fingertip pulse oximetry, heart rate, breathing rate, blood pressure, and electrocardiography. Abnormal blood pressure will be defined as low <90/60 mmHg and high >140/90 mmHg. Abnormal heart rate will be defined as <60 or >100 bpm. Abnormal breathing rate will be defined as <10 cycles/min or >20 cycles/min. Low oxygen saturation will be defined as levels <95%. Collateral symptoms such as nausea and vomiting will be managed using ondansetron 8 mg sublingually (SL). Individuals presenting episodes of anxiety, hallucinations, or intense dissociative effects will be given clonazepam SL 0.25 mg. An emergency team will be deployed in the event of serious acute events such as heart arrhythmia or prolonged hypertension. Patients will be placed under observation in a quiet comfortable environment and will be cleared to leave after 2 h, accompanied by a competent adult.

### Blinding

The researchers performing the clinical monitoring and application of the outcomes will be blinded, and the study participants will also be blinded. A nurse, not involved in data collection, will distribute the medications and apply the injections according to the randomization process.

### Sample size calculation

The sample size was calculated based on a similarly designed RCT (45). Assuming a statistical power of 90% with a clinically significant minimum effect of 0.5 (Cohen's d) and alpha 5%, a total of 88 subjects will be needed (44 in the EG and 44 in the CG). Given this is a long-duration study, a drop-out rate of 10–20% was estimated.

### Statistical analysis

All analyzes will be performed using the Statistical Package for the Social Sciences-SPSS® (82). Specific and sociodemographic variables will be expressed generally and between intervention groups in relative and absolute frequencies, means, medians, standard deviation, and 95% confidence interval. Continuous variables will be evaluated by the *T*-student or U-Mann Whitney-test according to their distribution and the qualitative ones by the *X*^2^-test. The distribution of variables will be evaluated using the Shapiro Wilk. The global significance level adopted is 0.05. To determine the effects of intervention throughout the study (main outcome and other scales), repeated measures ANOVA (two-way) will be applied, considering the temporal effect of visits (v-0 to v-final) and the intervention effect (EG × CG). In identifying the significant interaction effect, one-way repeated measures ANOVA test or its non-parametric alternative (Friedmann test) will be performed to verify that the effect is independent of treatment. Contingency tables and *X*^2^-tests will be applied to compare responders and non-responders in the intervention groups. Analysis using intent-to-treat (ITT) will be employed to assess the results (83). Vital signs will be assessed using two repeated measures ANOVA (two-way). First, considering the effect of the intervention immediately (3 evaluations at each visit) and the second, evaluating the effect of the intervention on VS over time (acute and maintenance treatment).

### Cognitive assessments

Neuropsychological tests will be applied at P0 and upon conclusion of PIII: to evaluate changes resulting from treatments:

**(1) Digit span (subtest of Wechsler Intelligence Scale):** measures attention span capacity and working memory; **(2) Wisconsin Card Sorting Test-64:** evaluate mental flexibility and ability to form abstract concepts in rapidly changing situations; **(3) Stroop Color-Word Test** (84): analyzes the maintenance of inhibitory control, from the suppression of the usual response in favor of an unusual response; **(4) Wechsler Abbreviated Scale of Intelligence (WASI):** the IQ is obtained from the sum of the gross results of the subtests converted to results weighted according to the individual's age (85); **(5) Verbal Fluency Test:** measures the ability to spontaneously produce words under semantic restriction and mind control (86); **(6) Rey's Complex Figure:** analyzes the visual-constructive ability, the ability to plan and problem-solving strategies (87) and; **(7) Trail-Making Test:** The purpose of this test is to assess alternate attention.

### Reasons for withdrawal or termination

Subjects who meet one or more of the criteria outlined below will be withdrawn from the study:

1. Intolerance to medications;
2. Abnormal results on lab tests;
3. Serious adverse event;
4. The above-mentioned items will be assessed individually by the team according to medical criteria. Prolonged arterial hypertension, serious allergies, abnormal heart rate, or worsening of pre-existing diseases, among others, will be checked. Manic switching, imminent risk of suicide, attempted suicide, severe psychotic symptoms, aggressiveness, etc. will constitute grounds for study withdrawal.
5. Relapse or lack of response in Phases II or III. Lack of response will be defined as reduction or maintenance of scores on the index scale of ≤25%, whereas relapse will be the attainment of scores <50% in individuals who had achieved remission or response during the study.
6. Two consecutive visits missed;
7. Withdrawal of consent for the study.

Patients who discontinue the study shall be given guidance to continue outpatient psychiatric treatment.

## Discussion

We describe a protocol for an RCT to assess the efficacy, safety, and tolerability of ketamine vs. active treatment in the acute and maintenance stages of TRD. This study represents the first such trial with this design to date. A total of 12 applications of ketamine IM (0.75 mg/kg) will be administered in the active phase and 24 in the maintenance phase to volunteers randomized into the EG. The sample size for the study was determined by drawing on a similar study found in the literature. The objectives of the planned study, besides the primary outcome of improving symptoms (as measured by the MADRS), is to assess the safety (VS) and tolerability of ketamine at sub-anesthetic doses and side effects, such as the development of depressive thoughts, functioning, quality of life, and neuropsychological effects, in addition to evaluating treatment efficacy in the acute and maintenance phases. The anti-depressant effects of ketamine appear to be dose-dependent and over the last 20 years have been studied using “sub-anesthetic” doses (0.5–1 mg/kg) for the treatment of depression (51). For this trial, it was decided to use ketamine at a dose that lies in the middle of this range (0.75 mg/kg), together with an administration route (IM) that provides similar bioavailability as IV, the most studied route to date. Studies comparing the different routes for ketamine in depression remain incipient (58). Although TRD is an amalgam of modern psychiatry, the impact of the disease and its myriad symptoms remain a major public health problem (19, 20). The issues involved in the efficacy and safety of ketamine IM include the wide use and accessibility of this low-cost substance, known to medicine for decades, yet lacking a clear prescription in current guidelines. The rapid anti-depressant and anti-suicidal action of the drug, together with its glutamatergic neurotransmitter regulation, has led to the recognition of ketamine as a new paradigm in modern psychiatry (34). Patients with compensated clinical comorbidities are to be included in the sample, conferring greater external validity to the findings. The study described must be interpreted according to its limitations regarding generalization of results: sample size, criteria for eligibility (exclusion of individuals with other decompensated psychiatric and clinical comorbidities). Difficulties blinding to ketamine given its dissociative effects, despite the various measures taken to safeguard this bias, may constitute a further limitation.

## Conclusions

The study will investigate the efficacy, safety, and tolerability of the use of ketamine IM in the treatment of TRD *via* a double-blind, parallel-group randomized, placebo-controlled trial. Clinical information, monitoring to control vital signs and standardized scales will help further understanding of the relationship of the disease with ketamine use. Future studies investigating the potential benefits of repeated ketamine infusions, non-parenteral administration alternatives, the safety of long-term use, reduced potential for abuse, and alternatives with fewer systemic effects can contribute to the management of TRD, a chronic and complex disease.

## Data availability statement

The original contributions presented in the study are included in the article/supplementary material, further inquiries can be directed to the corresponding author.

## Ethics statement

The studies involving human participants were reviewed and approved by Research Ethics Committee of the University of São Paulo (Permit No. 2.530.851). The patients/participants provided their written informed consent to participate in this study.

## Author contributions

Study conception and design: MC, FF, AC, and RM. Drafting of the manuscript: MC and RM. Statistic review: AG. Study execution: MC, DM, MAS, MSS, VA, JA, and AT. All authors contributed to the article and approved the submitted version.

## Funding

This work was supported by Research Support Fund (Institute and Department of Psychiatry).

## Conflict of interest

The authors declare that the research was conducted in the absence of any commercial or financial relationships that could be construed as a potential conflict of interest.

## Publisher's note

All claims expressed in this article are solely those of the authors and do not necessarily represent those of their affiliated organizations, or those of the publisher, the editors and the reviewers. Any product that may be evaluated in this article, or claim that may be made by its manufacturer, is not guaranteed or endorsed by the publisher.
